# A Multi-Omics Analysis of an Exhausted T Cells’ Molecular Signature in Pan-Cancer

**DOI:** 10.3390/jpm14070765

**Published:** 2024-07-18

**Authors:** Christos Rigopoulos, Ilias Georgakopoulos-Soares, Apostolos Zaravinos

**Affiliations:** 1 Department of Life Sciences, School of Sciences, European University Cyprus, Nicosia 2404, Cyprus; cr211316@students.euc.ac.cy; 2 Cancer Genetics, Genomics and Systems Biology Laboratory, Basic and Translational Cancer Research Center (BTCRC), Nicosia 1678, Cyprus; 3 Institute for Personalized Medicine, Department of Biochemistry and Molecular Biology, The Pennsylvania State University College of Medicine, Hershey, PA 17033, USA; izg5139@psu.edu

**Keywords:** exhausted T cells, pan-cancer, immunotherapy, gene expression, methylation, immune infiltration, drug sensitivity, CD8+ T cells, molecular signature

## Abstract

T cells are essential tumor suppressors in cancer immunology, but their dysfunction induced by cancer cells can result in T cell exhaustion. Exhausted T cells (Tex) significantly influence the tumor immune environment, and thus, there is a need for their thorough investigation across different types of cancer. Here, we address the role of Tex cells in pan-cancer, focusing on the expression, mutations, methylation, immune infiltration, and drug sensitivity of a molecular signature comprising of the genes HAVCR2, CXCL13, LAG3, LAYN, TIGIT, and PDCD1across multiple cancer types, using bioinformatics analysis of TCGA data. Our analysis revealed that the Tex signature genes are differentially expressed across 14 cancer types, being correlated with patient survival outcomes, with distinct survival trends. Pathway analysis indicated that the Tex genes influence key cancer-related pathways, such as apoptosis, EMT, and DNA damage pathways. Immune infiltration analysis highlighted a positive correlation between Tex gene expression and immune cell infiltration in bladder cancer, while mutations in these genes were associated with specific immune cell enrichments in UCEC and SKCM. CNVs in Tex genes were widespread across cancers. We also highlight high LAYN methylation in most tumors and a negative correlation between methylation levels and immune cell infiltration in various cancers. Drug sensitivity analysis identified numerous correlations, with CXCL13 and HAVCR2 expressions influencing sensitivity to several drugs, including Apitolisib, Belinostat, and Docetaxel. Overall, these findings highlight the importance of reviving exhausted T cells to enhance the treatment efficacy to significantly boost anti-tumor immunity and achieve better clinical outcomes.

## 1. Introduction

Cancer is a complex and heterogenous disease, and this poses challenges for its effective diagnosis and treatment [1,2]. It arises from successive mutations that alter cellular function and other factors such as chemical compounds, viruses, bacteria, and radiation. Oncogenes involved in cell division and growth also contribute to cancer by driving constant cell proliferation [3,4].

In recent times, targeting the immune system with immunotherapy has emerged as a promising strategy against cancer [5]. Immune checkpoint inhibitors (ICI), such as anti-CTLA-4, anti-PD1, and anti-PDL1 monoclonal antibodies (mabs), have shown notable success [6,7]. The adaptive immune system, regulated by lymphocytes, includes CD8+ T cells, which are key effectors in anti-tumor immunity [8]. These cells, developed in the thymus, recognize antigenic peptides presented by MHC class I molecules, initiating an immune response [9]. Persistent antigen exposure in cancer, however, can lead to T cell exhaustion, marked by loss of effector functions and upregulation of inhibitory receptors. What makes these cells different is the gradual loss of effector functions, the expression of multiple inhibitory receptors, metabolism dysregulation, poor memory recall response, and homeostatic proliferation. During exhaustion, CD8+ T cells are the first cells that lose some of their functions [10,11,12,13]. Dysfunctional or exhausted CD8+ T cells mainly lose their proliferative capacity either because of an intrinsic block or because their high inhibitory expression suppresses T cell activation. Various markers have been described for exhausted T cells (Tex), including LAG3, PDCD1, LAYN, CTLA4, and HAVCR2, among others [14]. In general, the functions that are absent in the preliminary (early dysfunctional) stages are IL-2 production, high proliferative capacity, and ex vivo killing [15]. Recently, T cell exhaustion has been associated with the tumor microenvironment (TME) [14]. From different studies, a signature characterizing Tex cells could be composed of the genes HAVCR2, LAG3, LAYN, PDCD1, TIGIT, and CXCL13 [14,16,17,18].

HAVCR2 (also known as T cell immunoglobulin and mucin-containing protein, or Tim-3) is an inhibitory receptor expressed on various immune cells, including T cells, natural killer (NK) cells, and dendritic cells. Tim-3 is involved in regulating immune responses, maintaining immune tolerance, and preventing autoimmune diseases. It plays a crucial role in T cell exhaustion, particularly in chronic infections and cancer, where its upregulation can lead to diminished T cell function and persistence of the disease [19,20].

CXCL13 is a chemokine that specifically binds to the chemokine receptor CXCR5. CXCL13 is primarily involved in the organization of B cell follicles in secondary lymphoid tissues. It plays a critical role in the homing of B cells and T follicular helper (Tfh) cells to lymphoid follicles, facilitating effective immune responses. Its expression is associated with the formation of tertiary lymphoid structures within tumors, contributing to the local immune microenvironment [21].

LAG3 (lymphocyte-activation gene 3) is an immune checkpoint receptor similar to CD4, binding MHC class II molecules. LAG3 negatively regulates T cell proliferation, activation, and homeostasis. It is expressed on activated T cells, NK cells, and regulatory T cells (Tregs). Like other inhibitory receptors, LAG3 contributes to T cell exhaustion in chronic diseases and cancer, making it a target for cancer immunotherapy.

LAYN (Layilin) is a C-type lectin-like receptor. While LAYN’s precise function in immunity is less well characterized compared to other genes in this signature, it has been implicated in the regulation of cell adhesion and migration. Recent studies suggest that LAYN may also play a role in T cell exhaustion and the suppression of immune responses within the TME [22,23].

TIGIT (T cell immunoreceptor with Ig and ITIM domains) is an inhibitory receptor expressed on T cells and NK cells. It competes with the co-stimulatory receptor CD226 for binding to the same ligands (CD112 and CD155). By doing so, it inhibits T cell and NK cell-mediated cytotoxicity and cytokine production. The expression of TIGIT is associated with T cell exhaustion in cancer and chronic infections, contributing to immune evasion by tumors [24].

PDCD1, or PD-1 (programmed cell death protein 1), is an immune checkpoint receptor expressed on T cells, B cells, and myeloid cells. PD-1 interacts with its ligands, PD-L1/2, to inhibit T cell activation and proliferation. This pathway is crucial for maintaining peripheral tolerance and preventing autoimmunity. In the context of cancer, the PD-1/PD-L1 interaction leads to T cell exhaustion, allowing tumors to evade immune surveillance. Blocking this pathway with antibodies (e.g., Pembrolizumab, Nivolumab, Cemiplimab, Atezolizumab, Durvalumab, etc.) has become a successful strategy in cancer immunotherapy [25,26,27,28,29,30,31].

Here, we explore the expression profile, mutations, drug resistance, and immune infiltration of the Tex signature in pan-cancer using bioinformatics analysis. Our findings support the idea of reviving exhausted T cells to enhance the treatment efficacy to significantly boost anti-tumor immunity and achieve better clinical outcomes.

## 2. Materials and Methods

### 2.1. Data Collection and Process

**Figure 1:**
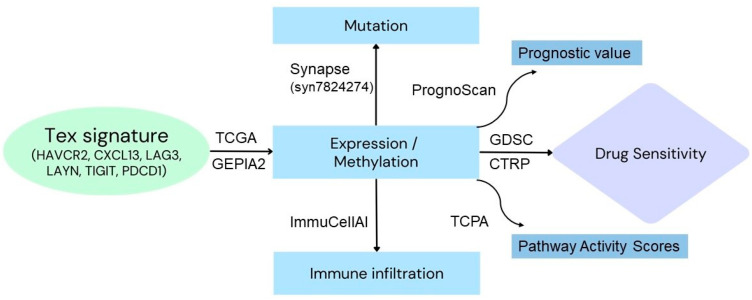
Workflow of the study. The genes HAVCR2, CXCL13, LAG3, LAYN, TIGIT, and PDCD1 were used to construct the Tex signature, according to previous publications. mRNA expression, gene mutation and methylation data were extracted from the TCGA and Synapse project. The mRNA expression of the Tex signature was also analyzed using GEPIA2. Reverse phase protein array (RPPA) data from TCPA were used to evaluate the pathway activity scores of 10 cancer-related pathways. ImmuCellAI was used for immune infiltration analysis. Data for drug sensitivity analysis were extracted from the GDSC and CTRP databases.

The TCGA mRNA expression, CNV, and methylation data were extracted from the UCSC Xena (https://xena.ucsc.edu/, accessed on 1 March 2024) [32]. The TCGA SNV data were obtained from Synapse (syn7824274) [33], while the drug sensitivity data were collected from the GDSC and CTRP databases. Samples with a competing risk for death from cancer were excluded (for DSS and DFI data). The mRNA expression data were RSEM normalized to remove batch effects [34,35]. All data were extracted as previously described in detail [36], and a general flowchart of the methodology that we followed is depicted in Figure 1.

### 2.2. Differential Expression of the Tex Signature

We investigated the differential expression of the Tex signature across 14 cancer types (bladder cancer, BLCA (n = 19); breast cancer, BRCA (n = 114); colon adenocarcinoma, COAD (n = 26); esophageal carcinoma, ESCA (n = 11); head and neck squamous cell carcinoma, HNSC (n = 43); kidney chromophobe, KICH (n = 25); kidney renal clear cell carcinoma, KIRC (n = 72); kidney renal papillary cell carcinoma, KIRP (n = 32); liver hepatocellular carcinoma, LIHC (n = 50); lung adenocarcinoma, LUAD (n = 58); lung squamous cell carcinoma, LUSC (n = 51); prostate adenocarcinoma, PRAD (n = 52); stomach adenocarcinoma, STAD (n = 32); and thyroid carcinoma, THCA (n = 59). The *p*-value was estimated by the *t*-test method and further adjusted by FDR. FDR values ≤ 0.05 were considered statistically significant.

We categorized tumor samples into high and low Tex expression groups and explored overall survival (OS), progression-free survival (PFS), disease-specific survival (DSS), and disease-free interval survival (DFI), as prognostic indicators. Cox proportional hazards models and log-rank tests were used for the analysis, and the results were presented using Kaplan–Meier curves.

To evaluate the candidacy of the genes in our Tex signature as potential tumor markers and therapeutic targets, we used PrognoScan (https://www.prognoscan.org/, accessed on 1 April 2024). PrognoScan is a large collection of publicly available cancer microarray datasets with clinical annotation, as well as a tool for assessing the biological relationship between gene expression and prognosis. The tool employs the minimum *p*-value approach for grouping patients for survival analysis that finds the optimal cutpoint in continuous gene expression measurement without prior biological knowledge or assumption and, as a result, enables systematic meta-analysis of multiple datasets.

In addition, we explored differences in gene expression of the Tex signature using GEPIA2 [37]. GEPIA2 uses a standard pipeline for the analysis of data from the TCGA and GTEx databases.

We also investigated changes in Tex expression across different tumor subtypes using the Wilcoxon and ANOVA tests. In addition, we explored different stage types by analyzing data from 9478 tumor samples across 27 cancer types, as previously described [36]. Pathologic, clinical, and Masaoka stages classify samples into stages I, II, III, and IV, while the IGCCCG classifies samples into good, intermediate, and poor. Trend analysis was conducted using the Mann–Kendall trend test.

In addition, we measured the pathway activities between high and low Tex gene expression as defined by their median pathway activity scores (PAS). Reverse phase protein array (RPPA) data from TCPA (https://www.tcpaportal.org/tcpa/, accessed on 15 April 2024) were utilized to evaluate the pathway activity scores of 10 cancer-related pathways. Differences in PAS between Tex-high and Tex-low expression subgroups were analyzed using *t*-test. *p*-values were FDR-adjusted, with a significance threshold set at 0.05.

### 2.3. Immune Infiltration and Tex Gene Expression Patterns in Tex Cells

For immune infiltration, we analyzed 4950 samples from 33 cancer types [34,35]. The evaluation of the infiltration of 24 immune cells was achieved using the Immune Cell Abundance Identifier (ImmuCellAI) (http://bioinfo.life.hust.edu.cn/ImmuCellAI/#!/, accessed on 1 May 2024). We correlated the Tex signature expression with the immune cells’ infiltrates using the Spearman’s test. Statical significance was set at FDR ≤ 0.05.

### 2.4. Immune Infiltration and Tex Gene Mutations (CNVs, SNVs)

Using the Wilcoxon test, we further assessed the differences in immune cell infiltrates between mutated (SNV) and wild-type (WT) genes. Subsequently, we explored the correlation between copy number variations (CNVs) affecting the Tex signature genes and immune cell infiltrates using Spearman’s test.

### 2.5. Immune Infiltration and Methylation

We correlated methylation affecting the Tex signature with immune cells’ infiltrates using the Spearman’s test and adjusted *p*-values using FDR.

### 2.6. Tex Gene Mutation

We explored single nucleotide variations (SNVs) affecting the Tex signature, across 10,234 samples from 33 cancer types [34,35]. We focused on the presence of deleterious mutations such as missense and nonsense mutations, frame-shift insertions/deletions, splice-site mutations, and in-frame insertions/deletions. We also evaluated survival differences between mutant and wild-type groups using the R package “survival”, the Cox proportional hazards model, and log-rank tests. Co-mutations were linked to cancer clinical outcomes. CNV data from 11,495 samples in TCGA were downloaded and analyzed for significantly amplified or deleted genomic regions with GISTIC2.0.

We also correlated Tex CNVs with gene expression patterns in Tex using Spearman’s test. The *p*-value was adjusted by FDR.

We further divided samples into wild-type (WT), amplified (Amp.), and deleted (Dele.). The survival differences between the groups were evaluated using the log rank test and the package “survival” in R.

### 2.7. Differential Methylation

We then explored the differential methylation between tumor and normal sample groups by extracting data (beta values) from the Illumina Human Methylation 450 K. Multiple methylation tags were used for each site. A Spearman’s test was used to identify the correlation between gene expression patterns in Tex and methylation levels.

Tumor samples were divided into high and low Tex methylation groups based on the median methylation level, and survival differences between these groups were assessed using the “survival” package in R.

### 2.8. Correlation between the Tex Signature and Drug Sensitivity

We collected the inhibitory concentration (IC50) of a large number of small molecules in various cell lines and their corresponding gene expression patterns in Tex from Genomics of Drug Sensitivity in Cancer (GDSC). We correlated the gene expression patterns in Tex and Drug IC50 using Pearson’s test and adjusted the *p*-value using FDR. We also selected the IC50 from 481 compounds in 860 cell lines and its matching gene expression patterns in Tex from the Genomics of Therapeutics Response Portal (CTRP v2) and analyzed them using Pearson’s correlation test.

## 3. Results

### 3.1. Differential Tex Expression in Pan-Cancer

**Figure 2:**
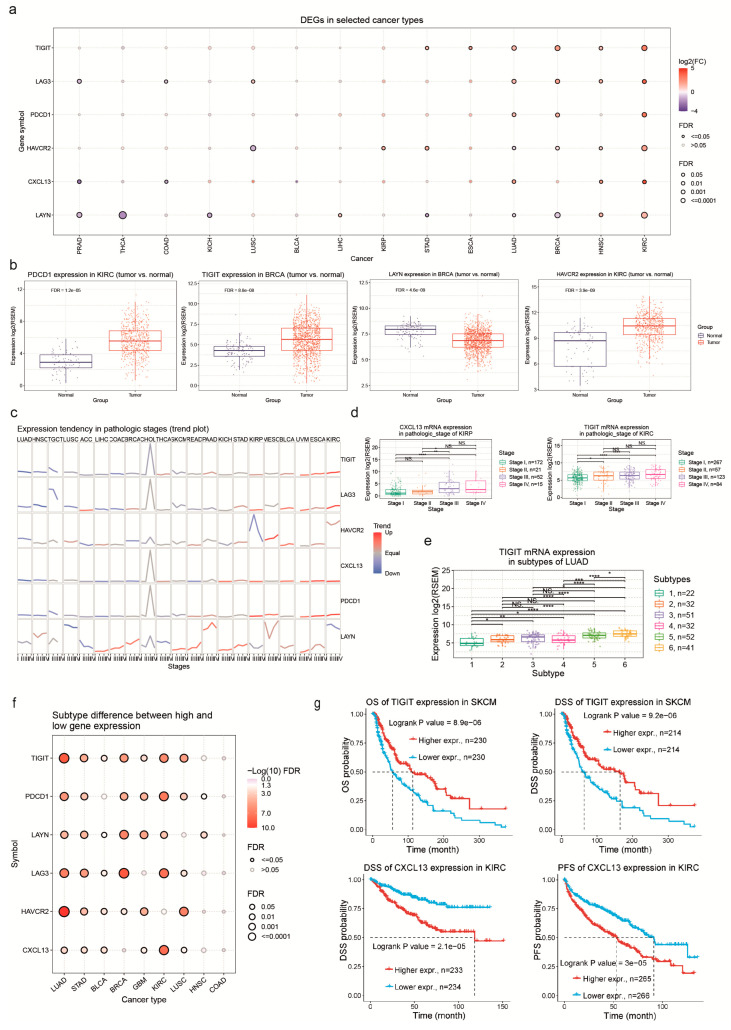
(**a**) The bubble plot shows the fold change of *HAVCR2*, *CXCL13*, *LAYN*, *TIGIT*, *LAG3*, and *PDCD1* across 14 tumors. (**b**) TIGIT and LAYN mRNA expression in BRCA and *PDCD1* and *HAVCR2* expression in KIRC, compared to normal tissues. (**c**) The trend plot depicts the expression of *TIGIT*, *HAVCR2*, *CXCL13*, *LAYN*, *PDCD1*, and *LAG3* genes across different cancer stages. (**d**) *CXCL13* and *TIGIT* expression in pathologic stages of KIRC and KIRP. *, *p* < 0.05; **, *p* < 0.01; ****, *p* < 0.001 and NS, not significant (**e**) The boxplots to the right depict examples of differential expression of *PDCD1* and *TIGIT* expression in molecular subtypes of LUAD. (**f**) The correlations between different cancer subtypes and *TIGIT*, *HAVCR2*, *CXCL13*, *LAYN*, *LAG3*, and *PDCD1* expression. (**g**) Examples of PFS, OS, and DSS differences between high and low *CXCL13* and *TIGIT* expressing tumors in KIRC and SKCM.

We initially explored the expression of the Tex signature genes (*LAG3*, *TIGIT*, *LAYN*, *PDCD1*, *HAVCR2*, and *CXCL13*) in pan-cancer. We found higher expression levels of all Tex-related genes in KIRC and LUAD, compared to their corresponding normal tissue (Figure 2a). Additionally, *LAYN* expression was lower mainly in THCA but also in KICH and BRCA (Figure 2b). *TIGIT* and *LAG3* expression was equally higher in LUAD and BRCA. We finally figured out that *HAVCR2* expression was slightly low in LUSC. Trend analysis also showed differences in *LAYN* expression between pathological and clinical stages in various cancers, such as LUSC and BRCA (Figure 2c). Visible differences were observed between different cancer stages in KIRP, according to *CXCL13* mRNA levels. We also found Tex expression differences between clinical and pathologic stages in KIRC (Figure 2d). In addition, we found a correlation between *TIGIT*, *HAVCR2*, *LAG3*, and *PDCD1* expression and different subtypes in LUAD (Figure 2e,f).

### 3.2. Correlation between Gene Expression Patterns in Tex and Patient Survival in Pan-Cancer

We aimed to understand the prognostic value of the Tex signature genes in pan-cancer. To achieve this, we analyzed differences in patient survival between high and low Tex-expressing tumors. Indeed, there was a correlation between Tex gene expression and patient survival. We observed that in SKCM, lower *TIGIT* (and other Tex genes) expression correlated with worse patient survival. In contrast, in KIRC, there was a reverse pattern, where higher Tex expression correlated with worse patient survival (Figure 2g).

**Figure 3:**
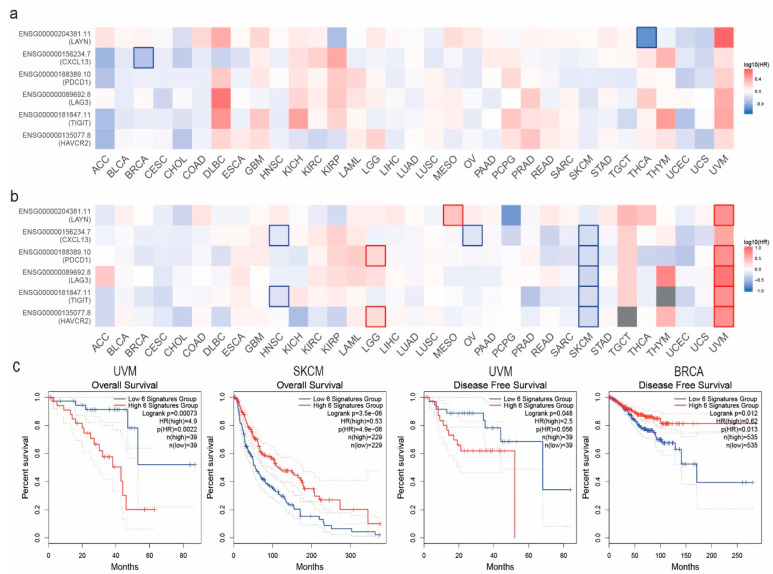
Survival map showing the overall survival (OS) (**a**) and disease-free (DFS) survival (**b**) of the genes in the Tex cell signature in pan-cancer. The Kaplan–Meier curves below show the (OS) and (DFS) differences between high and low Tex gene-expressing groups in uveal melanoma (UVM), skin melanoma (SKCM), and breast cancer (BRCA) (**c**). An FDR-adjusted *p* < 0.05 was set as threshold for significance. Bold red and blue lines represent the 50th percentiles of high and low signature groups, respectively. Dashed lines represent the 25th and 75th percentiles.

Using GEPIA2, we investigated the overall and disease-free survival maps (OS, DFS) of the Tex signature in pan-cancer (Figure 3a). Our results demonstrated that in UVM, DLBC, KICH, and KIRC, the OS and DFS were higher in high Tex-expressing groups. In contrast, in PCPG, PRAD, and SKCM, the low Tex-expressing groups indicated a higher overall survival rate than the high Tex-expressing groups. In addition, in ACC, CESC, CHOL, and LIHC, the low Tex-expressing groups showed a greater DFS compared to the high Tex-expressing ones (Figure 2b). Furthermore, we tried to predict the prognostic risk of the Tex signature in pan-cancer using PrognoScan. Prognoscan utilizes public resources in full and provides a major collection of cancer microarray datasets with clinical annotation. It investigates the relationship between gene expression and patient prognosis, like OS and DFS. The results indicated that the gene signature is associated with the prognosis in a variety of tumors. For example, *CXCL13* has a protective prognostic action (*p* < 0.05, HR < 0) in breast cancer and skin melanoma. At the same time, it is an adverse prognostic factor (*p* < 0.05, HR > 0) in follicular lymphoma and bladder cancer (transitional cell carcinoma). *HAVCR2* indicated a simultaneous adverse and protective prognostic action in lung cancer (adenocarcinoma) and an adverse prognostic action in breast and colorectal cancer. *LAG3* showed a protective effect in AML, ovarian cancer, and breast cancer, while in bladder cancer (transitional cell carcinoma) and breast and brain cancer (glioma), it presented an adverse prognostic role. Moving on, *LAYN* was mainly considered an adverse prognostic factor in brain cancer (glioma), colorectal, and ovarian cancer, while it indicated a positive prognostic role in breast cancer. The TIGIT gene presented a significant prognostic action only in uveal melanoma. Finally, *PDCD1* exhibited a prognostic role in a wide variety of tumors. It had an adverse prognostic role in DLBCL, breast cancer, lung adenocarcinoma, and ovarian cancer, while it was considered to have a protective prognostic factor in colorectal cancer, uveal melanoma, and skin melanoma.

### 3.3. Pathway Activity in Pan-Cancer

**Figure 4:**
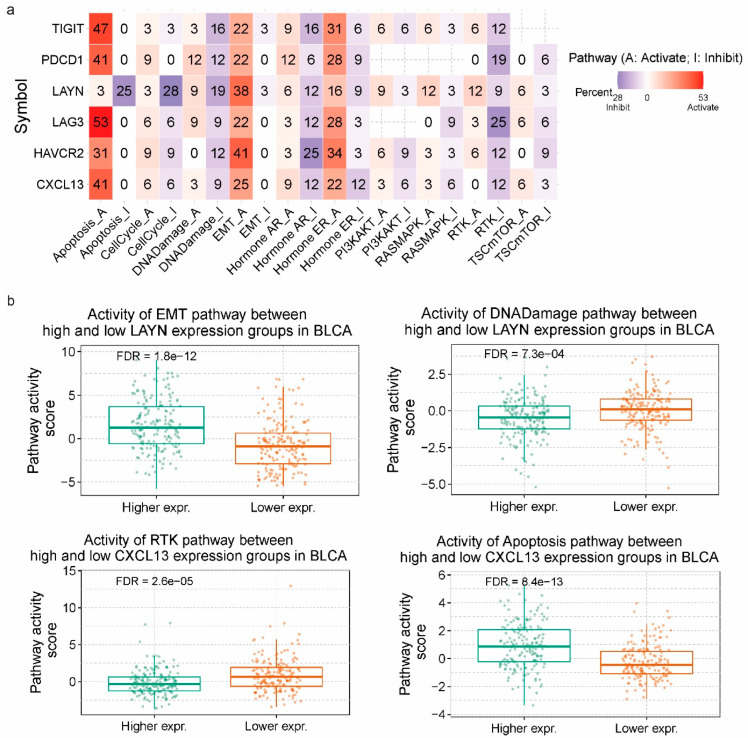
(**a**) The percentage (%) of tumors in which the expression of *TIGIT*, *CXCL13*, *HAVCR2*, *LAYN*, *LAG3*, and *PDCD1* may affect the activity of 10 cancer-related pathways. The red color stands for activation of the pathway, while blue shifts towards its inhibition. The number in each cell represents the % of cancer types in which each of the 6 genes in the Tex signature presented a significant effect (either inducing or inhibitory). (**b**) Pathway activity scores (PAS) in high and low *LAYN* (and *CXCL13*)-expressing urinary bladder cancers (BLCA).

We then set out to investigate the differences in the activity of 10 cancer-related pathways between high and low Tex-expressing tumors. The heat map depicts the percentage (%) of cancers in which each gene has an effect on the pathway among certain tumors. We found an inducing effect of TIGIT on apoptosis (47%), EMT (22%), and ER (31%). That means that TIGIT activates the apoptosis pathway in 47% of the selected cancers, EMT pathway in 22%, and ER in 31% of them. Moving on, an equally activating effect of LAG3 on apoptosis (53%), EMT (22%), and ER (28%). Regarding *HAVCR2*, we observed a strong inducing effect on apoptosis (31%), EMT (41%), and ER (34%), while we also saw an inhibitory effect on DNA damage (12%), AR (25%), and RTK (12%). LAYN indicated stronger repressive action on apoptosis (25%), cell cycle (28%), DNA damage (19%), and AR (12%). At the same time, it showed some inducing effects on EMT (38%) and ER (16%) (Figure 3a). For example, low LAYN-expressing BLCA tumors, had higher activity scores in the DNA damage pathway (FDR = 7.3 × 10^−4^), compared to the EMT pathway (FDR = 1.8 × 10^−12^), whereas, high-expressing LAYN tumors showed slightly better activity scores. Also, high CXCL13-expressing tumors showed a better activity score in the apoptosis pathway (FDR = 8.4 × 10^−13^). In contrast, low *CXCL13*-expressing tumors indicated a better activity score regarding the RTK pathway (FDR = 2.6 × 10^−5^) (Figure 4b).

### 3.4. Correlation between the Tex Signature Expression and Immune Infiltration in Pan-Cancer

**Figure 5:**
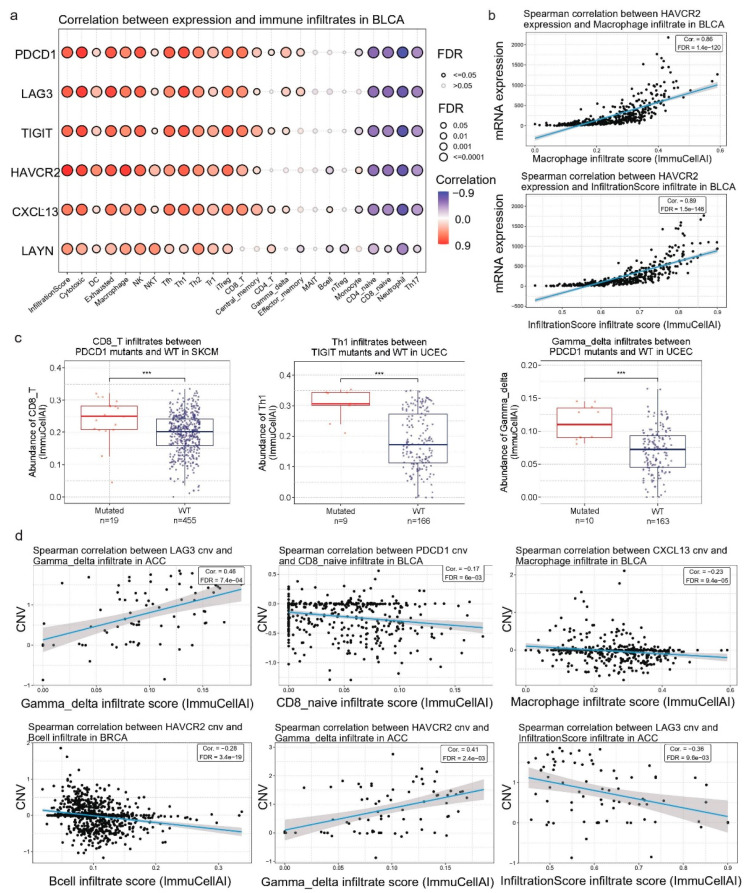
(**a**) Correlation between *HAVCR2*, *CXC13*, *TIGIT*, *LAYN*, *LAG3*, and *PDCD1* mRNA levels and immune infiltrates in BLCA. (**b**) CD8_T infiltrates between mutant and WT PDCD1 in SKCM. Gamma-delta infiltrates between mutant and WT *PDCD1* in UCEC and Th1 infiltrates between mutant and WT *TIGIT* in UCEC. (**c**) Spearman correlation between HAVCR2, infiltration score, and macrophages in BLCA. ***, *p* < 0.001. (**d**) Spearman’s correlations between *PDCD1*, *HAVCR2*, *LAG3*, and *CXCL13* CNVs and immune cell infiltrates in BRCA, BLCA, and ACC.

We then correlated the Tex signature’s expression with immune infiltration across different tumors. To achieve this, we measured the infiltrates of 24 immune cells in the tumors, using ImmuneCellAI. Overall, our results indicated a positive correlation between the Tex signature’s expression and the immune infiltration score in bladder cancer. There was also a positive correlation between the Tex expression and infiltration of cytotoxic, dendritic cells (DC), exhausted macrophages, NK, NKT, Tfh, and Th1/2, Tr1, and iTreg cells as well as a negative correlation with infiltration of neutrophils, CD4/8 naïve, and Th17 cells (Figure 5a,b).

### 3.5. Tex Genes’ Mutation Status and Correlation with Immune Infiltration in Pan-Cancer

**Figure 6:**
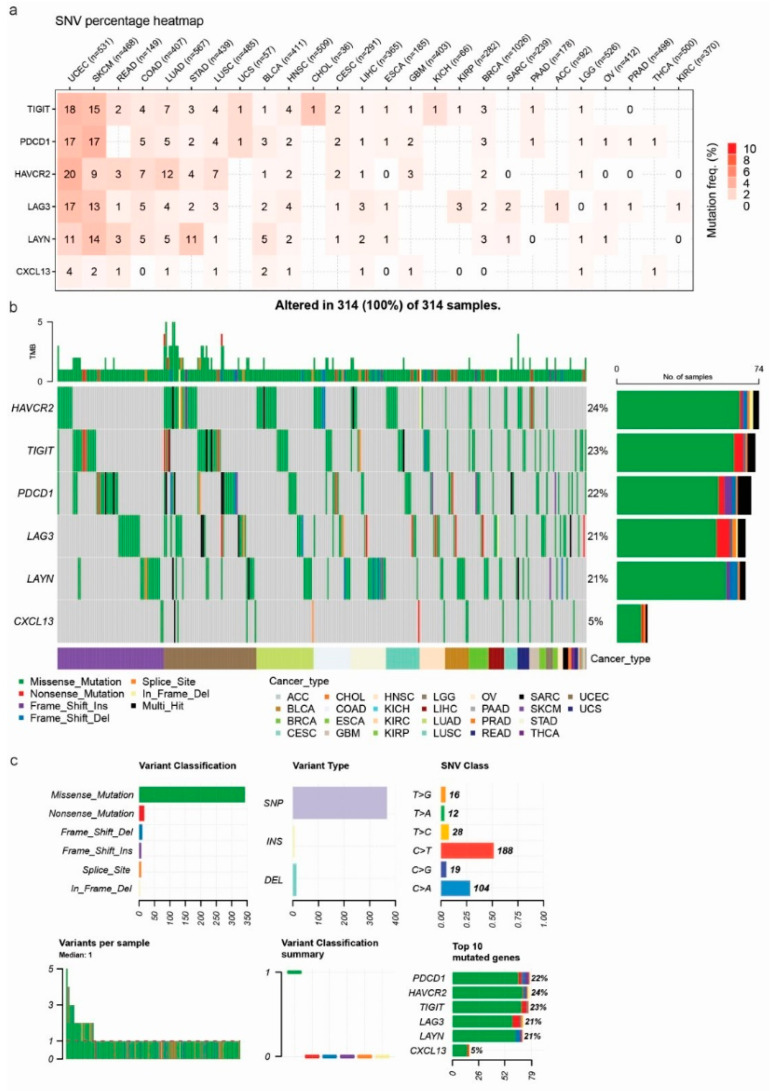
(**a**) SNV percentage heatmap of the Tex gene signature in pan-cancer. (**b**) Oncoplot depicting the Tex signature mutational profile in pan-cancer. Each column corresponds to a unique sample. The right-side bar plots depict the number of samples affected by mutations gene-wise, and the top-side bar plots the mutation number (TMB) per sample. The bottom annotation color bar distinguishes different cancer types. (**c**) The number of variants in each sample is displayed as a stacked barplot and variant types as a boxplot summarized by variant classification. Top: Variant classification, variant types (SNP, INS, and DEL), and SNV classes. Overall, SNVs were classified into six substitution classes (T>G, T>A, T>C, C>T, C>G, and C>A). Bottom: Variants per sample, variant classification summary, and ranking of the Tex signature according to their mutation rate.

Investigating mutations in genes involved in exhausted T cells is crucial because these mutations could reveal mechanisms underlying immune dysfunction and potential therapeutic targets for rejuvenating T cell responses in cancer. Understanding these genetic alterations could help develop strategies to enhance immune checkpoint therapies and improve patient outcomes. To this end, we explored the mutation rate of the Tex signature genes in pan-cancer, and we observed a 2–3% SNV mutation frequency of *PDCD1*, *LAG3*, *TIGIT*, *HAVCR2,* and *LAYN* mainly in UCEC and SKCM, with *HAVCR2* and *TIGIT* having the highest mutation frequencies (24% and 23% of samples, respectively). This was followed closely by *PDCD1*, *LAG3*, and *LAYN*, each presenting mutations in approximately 21–22% of samples. *CXCL13* showed the lowest mutation frequency, with only 5% of samples affected. The majority of mutations were missense mutations of C>T class. Interestingly, a significant degree of mutual exclusivity was pointed out in the mutations of the Tex genes, suggesting that mutations in these genes do not frequently co-occur within the same samples, as well as that each gene may play a distinct and non-redundant role in tumor biology and immune evasion mechanisms, potentially providing independent pathways for tumor survival and progression. The mutation prevalence and distribution among the Tex signature genes across multiple cancer types is shown in Figure 6.

We then set out to observe differences in immune cell infiltration between mutants (MTs) and WT tumors. Our results indicated a significant abundance of macrophages in WT TIGIT skin melanomas (*p* = 0.008), as well as an enrichment of neutrophils in mutant PDCD1 skin melanomas (*p* = 0.007). Macrophages are tumor-promoting cells acting by secreting cytokines, such as IL-6, IL-1β, and TNF-α. They bind to TNF receptors, activating the NF-kB pathway, which promotes cell proliferation and survival. The pro-inflammatory action of IL-6, supported by the JAK/STAT3 pathway, drives cell proliferation, differentiation, and apoptosis [38]. Neutrophils, on the other hand, express chemokine receptors CXCR1 and CXCR2, which are attracted by CXCR2 ligands, and they infiltrate the TME. Cancer cells express lots of chemokines and recruit neutrophils, which release reactive oxygen species (ROS), or proteases, and support cancer initiation. They also support tumor growth by promoting angiogenesis, as neutrophil depletion suppresses vessel formation [39,40,41,42].

Additionally, we found a significant enrichment of Th1 cells in TIGIT mutant UCECs and an abundance of gamma-delta (γδ) T cells in PDCD1 WT UCECs (Figure 5b). The role of Th1 cells is to induce macrophages and neutrophils. Thus, they are vital for host defense versus intracellular pathogens, like *M. tuberculosis.* γδ T cells are an important subset of “unconventional” T lymphocytes because they can recognize a wide range of antigens without the presence of MHC molecules. They can attack target cells through their cytotoxic activity or the activation of other immune cells. For example, in hematological malignancies, they use their cytotoxic activity against lymphoid leukemia cells associated with the expression of several NK receptors [43].

Overall, our results indicate a small percentage of Tex signature mutations in UCEC and SKCM being associated with the infiltration of specific immune cells in these tumors.

### 3.6. Correlation between Tex Signature CNVs and Immune Infiltration in Pan-Cancer

Following, we evaluated the percentage of heterozygous and homozygous CNVs of the Tex signature in pan-cancer. We found a large distribution of mainly heterozygous CNVs affecting the Tex genes across all tumor types, apart from THCA, THYM, and LAML. The highest percentages of CNVs were observed in ACC, OV, KICH, READ, LUAD, MESO, LUSC, HNSC, STAD, UCS, BRCA, ESCA, CESC, BLCA, SARC, and TGCT. We tried to analyze better the mechanisms underlying the abnormal gene expression patterns in Tex, by investigating the correlation between the CNVs affecting these loci and the expression of this signature, in pan-cancer. Our results indicated a wide variety of correlations between CNVs affecting the Tex genes and immune infiltrates in BLCA, BRCA, and ACC, among other tumor cancer types. For instance, in BLCA, we observed correlations between *PDCD1* CNVs and CD8 naive immune filtrates (Corr = −0.17, *p* = 5.16 × 10^−4^, FDR = 6.03 × 10^−3^), NK (Corr = 0.18, *p* = 2.35 × 10^−4^, FDR = 3.4 × 10^−3^), as well as CXCL13 CNVs with macrophages (Corr = −0.22, *p* = 3.69 × 10^−6^, FDR = 9.37 × 10^−5^). Regarding HAVCR2, we spotted correlations between CNVs and B cells (Corr = −0.28, *p* = 6.92 × 10^−21^, FDR = 3.38 × 10^−19^) in BRCA and gamma-delta (Corr = 0.4, *p* = 2.3 × 10^−4^, FDR = 2.4 × 10^−3^) in ACC. We also highlighted correlations between LAG3 CNVs with gamma-delta (Corr = 0.46, *p* = 2.41 × 10^−5^, FDR = 739 × 10^−4^) and infiltration score (Corr = −0.36, *p* = 1.27 × 10^−3^, FDR = 9.61 × 10^−3^) in ACC (Figure 5c,d).

Naïve CD8 T cells are found mainly in the circulation, spleen, and lymph nodes. They survey the entire body for DCs presenting cognate antigens that will result in their activation. Their existence has been described in depth in breast cancer. Macrophage infiltration is associated with a high vascular grade, reduced relapse-free survival, and decreased overall survival and serves as a prognostic biomarker of breast cancer. The immune infiltration score is a way of quantifying cell infiltration within cancers to predict prognosis and chemotherapy side effects. In addition, B cells can inhibit cancer development through the production of tumor-reactive antibodies [44].

### 3.7. Correlation between Methylation Affecting the Tex Signature and, Immune Infiltration in Pan-Cancer

**Figure 7:**
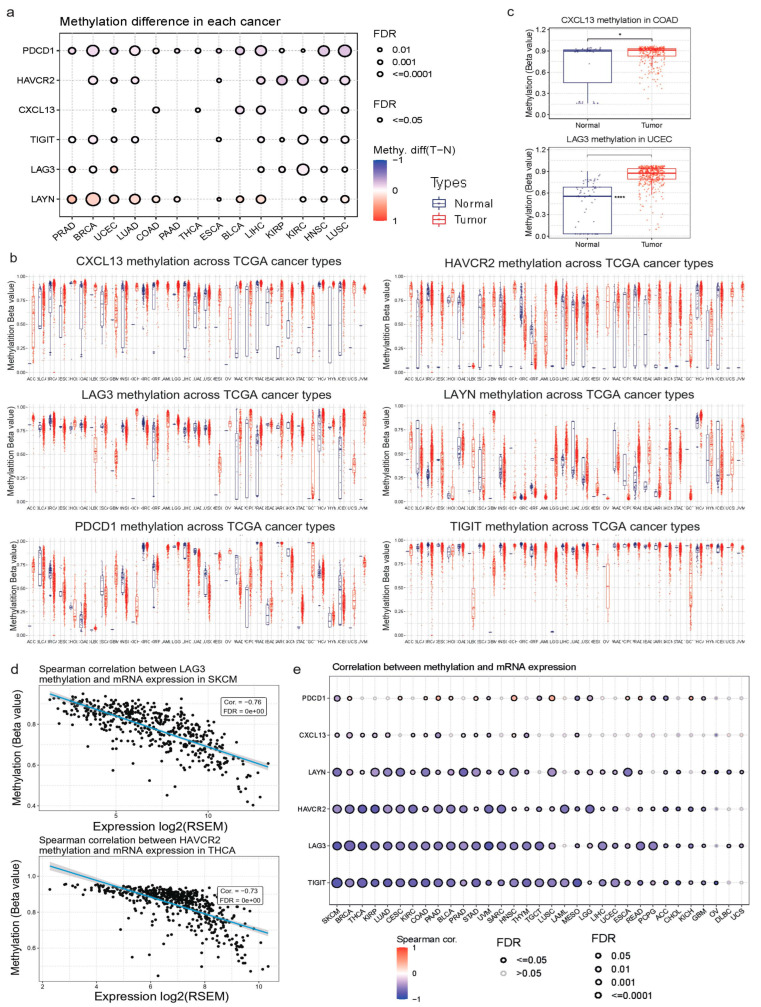
(**a**) Differential *CXCL13*, *HAVCR2*, *TIGIT*, *LAYN*, *LAG3,* and *PDCD1* methylation levels (beta values) between tumor and normal samples in pan-cancer. (**b**) Differential methylation levels of *LAYN* and *CXCL13* in COAD and *LAG3* in UCEC. (**c**) Spearman correlations between *LAG3* and *HAVCR2* mRNA levels in SKCM and THCA, respectively. *, *p* < 0.05; ****, *p* < 0.001 (**d**) Correlation between *HAVCR2*, *CXCL13*, *LAYN*, *TIGIT*, *LAG3*, and *PDCD1* and their gene expression in pan-cancer.

We investigated the methylation levels (beta values) of *TIGIT*, *CXCL13*, *LAYN*, *LAG3*, *PDCD1*, and *HAVCR2* across different cancer types. Our analysis revealed that *LAYN* methylation levels were high in most tumors. We also demonstrated that *PDCD1*, *HAVCR2,* and *CXCL13* had low methylation levels in the majority of the tumors examined. Regarding the *LAG3* methylation, it was not so clear compared to the two previous genes. Nevertheless, we observed some exceptions in MESO, SARC, TGCT, UCEC, and UVM (Figure 7a–c).

We then focused on exploring the correlation between the Tex genes‘beta values and immune cell infiltrates across different tumors. We observed that each gene (apart from *PDCD1* and *CXCL13*) exhibited a strong negative correlation between methylation and immune cell infiltrates in ACC, BLCA, BRCA, COAD, SKCM, UCEC, THYM PAAD, KIRP, and KIRC, among other cancer types. Moreover, we found that there is a strong negative correlation between *LAG3* and *HAVCR2* methylation and gene expression in SKCM and THCA, as well as in LUAD and KIRC (Figure 7d). Specifically, in SKCM, we found that *HAVCR2* methylation is strongly correlated with infiltrates of CD4 and CD8 T cells, central memory, cytotoxic, exhausted, gamma-delta, the Infiltration Score, monocytes, NK, neutrophils, Tfh, Th1, Th17, and iTreg cells. Respectively, in THCA, we found a correlation between *LAG3* methylation and infiltrates of CD4 and CD8 T cells, as well as CD4 and CD8 naïve cells, central memory, cytotoxic T cells, DCs, exhausted T cells, the infiltration score, MAIT, macrophages, neutrophils, Tfh, Th1, Th17, Tr1, and iTreg cells.

Overall, our findings indicate that the Tex signature might play a crucial role in specific tumors, such as THCA and SKCM. They also recommend that *HAVCR2* and *LAG3* are significantly associated with immune infiltration in these tumors.

### 3.8. Correlation between the Tex Signature’s Expression and Drug Sensitivity in Pan-Cancer

**Figure 8:**
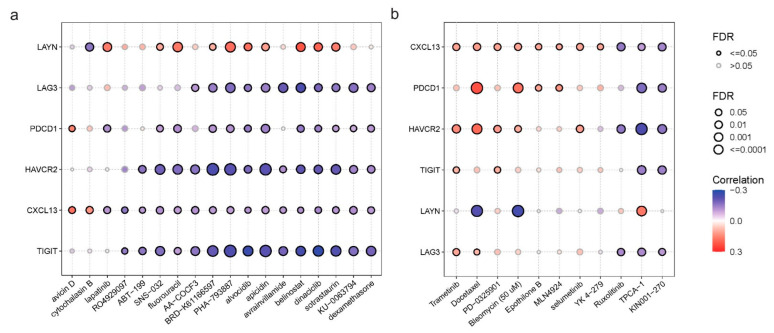
Correlation of HAVCR2, CXCL13, TIGIT, LAYN, LAG3, and PDCD1 expression levels with drug sensitivity (IC50) in pan-cancer, using the CTRP (**a**) and GDSC (**b**) databases. The color from red to blue depicts the correlation between each gene’s mRNA expression and IC50. Also, the bubble size represents the false discovery rate (FDR), with larger circles indicating stronger statistical significance. The color gradient indicates the direction and magnitude of correlation. Blue for negative correlation and red for positive correlation. Significant correlations (FDR < 0.05) are emphasized with bold outlines, highlighting the most critical interactions.

Finally, we gathered the IC50 of a wide variety of drugs among different cancer cell lines through the GDSC and CTRP databases and tried to explore the possible associations with the corresponding *CXCL13*, *HAVCR2*, *LAG3*, *TIGIT*, *PDCD1*, and *LAYN* mRNA values. Overall, we found a large number of correlations. For example, CXC13 mRNA levels were negatively correlated with sensitivity to various drugs, including, AA-COCF3, SNS-032, Apicidin, and KU-0063794, while they were positively associated with Avicin D and Cytochalasin B sensitivity in the CTRP database. Interestingly, HAVCR2 levels were negatively correlated with multiple drugs, such as Apitolisib, Belinostat, and Dacinostat, indicating that higher expression of HAVCR2 is associated with increased sensitivity to these drugs. We found the same result for TIGIT and LAG3. LAYN mRNA expression was positively correlated with Lapatinib, Fluorouracil, PHA-793887, Belinostat, and Dinaciclib sensitivity. Last, PDCD1 levels were positively correlated only with sensitivity to Avicin D (Figure 8a).

In the GDSC database, CXCL13 levels were positively correlated with sensitivity to specific drugs like Trametinib, indicating that higher expression of CXCL13 is associated with increased drug resistance. On the other hand, they were negatively correlated with Ruxolitinib, TPCA-1, and KIN001-270 sensitivity. PDCD1 showed a strong positive correlation with Docetaxel and Bleomycin (50 μM) and a less strong negative correlation with TPCA-1 and KIN001-270 sensitivity. Regarding LAG3 and TIGIT, we did not observe correlations worth mentioning. LAYN mRNA expression showed an extremely strong negative correlation with Docetaxel and Bleomycin (50 μM). It also exhibited a positive correlation with TPCA-1 sensitivity. Finally, HAVCR2 was positively correlated with docetaxel and negatively with TPCA-1 (Figure 8b).

## 4. Discussion

Dysregulation of exhausted T cells has been previously discussed in a substantial number of tumors. In the majority of studies and reports, the researchers have focused on each gene, separately. Here, we investigate in depth a newly suggested Tex signature in terms of its expression, mutation, correlation with pathways, immune infiltration, and patient survival in pan-cancer.

Immune checkpoint inhibition (ICI) therapies have gained a lot of attention, especially in patients who respond poorly either to radiotherapy or chemotherapy. ICIs have been tremendously effective in cancer treatment, as they block signals that allow cancer cells to avoid immune detection, providing long-term responses and giving hope to many cancer patients. The most widely tested blockades are against the PD-1/PD-L1 axis [45]. Anti-PD-1/PD-L1 blockades play a crucial role in anti-tumor immunity, as many signals in cancer cells can regulate them. The most vital signals are from pathways like PIK3/AKT, JAK/STAT3, NF-κB, and MAPK, all of which promote cell proliferation, survival, and apoptosis-related processes [46].

Epigenetic modifications have been shown to affect the expression of different immune checkpoints. For example, in colorectal cancer, DNA hypomethylation and two repressive histone marks, H3K9me3 and H3K27me3, were shown to be involved in the upregulation of CTLA-4 and TIGIT genes. Similarly, there is an abundance of H3K9me3 and H3K27me3 in the promoter regions of PD1 [47].

In another study, the promoter of PD-1 was also found to be hypermethylated, while PD-L1 was hypomethylated, in breast and colorectal cancers [47]. Furthermore, TIGIT was significantly hypomethylated in colorectal cancer. Remarkably, the promoter methylation status of LAG3, TIGIT, and PD-L1 was anti-correlated with gene expression in colorectal cancer [47].

Similarly, TIGIT expression was shown to be epigenetically regulated via DNA methylation in skin melanoma, suggesting that TIGIT DNA methylation and expression may serve as predictive biomarkers in the context of immunotherapies [48].

Sasidharan et al. also showed that both DNA and histone modifications are involved in the upregulation of PD-1, CTLA-4, TIM-3, and LAG3 in breast cancer, suggesting that these genes could be used as diagnostic/prognostic biomarkers and/or therapeutic targets as well [48].

TIGIT plays a dual role in immune resistance, as it restricts adaptive and innate immunity against malignancies, like lung and kidney cancers [49]. Response rates to ICIs can differ across cancer types and individual patients. This is due to the interaction between cancer cells and the immune system in the TME. One of the strategies that have been proposed to address this problem is double ICI therapy, i.e., by combining two inhibitory receptors at the same time to strengthen the anti-tumor immune response. TIGIT has emerged as one of the most promising candidates for co-inhibition with PD-1/PD-L1 in tumor immunotherapy. It has also been identified that TIGIT is correlated with T cell exhaustion and immunosuppressive effects in different cancer types [50,51,52,53]. Different studies have shown that TIGIT is overexpressed across different cancer types, such as KIRP, KICH, LUAD, and COAD [54,55]. This agrees with our results. Additionally, another study mentioned the positive correlation between TIGIT expression and the various stages of KIRP [56], which we corroborate. Moreover, we identified elevated levels of TIGIT in LUAD and BRCA, which agree with the conclusions of Han et al. (2020) [44]. In agreement with our findings, Wen et al. (2021) showed that TIGIT has a prognostic role in UVM. TIGIT has also been involved in improving CAR-T efficacy by inhibiting the TIGIT pathways [45].

The significance of the HAVCR2 gene is related to its ligand, TIM-3. TIM-3 has emerged as one of the future targets for immunotherapy. Therefore, the study of the HAVCR2 gene is important [19]. Analyzing our data, we show that HAVCR2 expression is increased in the majority of the different cancer types. One of the few exceptions is the decreased expression in LUSC. This result agrees with Li et al. (2022) [19], who conducted a similar analysis. In the same study, HAVCR2 was found to have a prognostic role in CESC, KIRC, SKCM, and osteosarcoma, and an adverse role in the prognosis of GBM, GBMLGG, LGG, PRAD, and UVM. We also showed that HAVCR2 was a protective factor in LUAD, while it has a worse prognosis in LUAD, BRCA, and COAD. We also found that there is a close relationship between HAVCR2 and the immune system, as we indicated that there is a strong correlation between the genes’ expression and immune infiltration and macrophages. This verifies that HAVCR2 may be a promising future gene to target. The same results and conclusions were recently reported by Xue et al. (2023) [57]. We further analyzed the mutation frequency of HAVCR2 in pan-cancer and found 2–3% in SKCM and UCEC. This agrees with Li et al. (2022), who found that the mutation rate of HAVCR2 in SKCM is 2.3% [58]. HAVCR2 has been found to be highly methylated in a majority of tumors, especially in melanoma [48,59]. DNA methylation is present in possibly each and every region of the genome. It can be predominantly found in the gene promoter region, usually within the CpG islands, and leads to the suppression of transcription [49,50]. HAVCR2 has been recently found to contain enrichment of methylation sites inside the 3′ UTR, suggesting that this region is a potent target for cancer therapy and a new biomarker for better diagnosis and prognosis of tumors [60]. Another region worth mentioning is the non-CpG island promoter region, which was found to involve gene expression despite the promoter region showing a decreased methylation level [61,62]. By studying the methylation of HAVCR2, we found that there is an exceeding methylation across different cancer types, such as ACC, CESC, COAD, GBM, KICH, LGG, MESO, OV, READ, SARC, SKCM, STAD, UCS, and UVM. In contrast, in testicular germ cell tumors (TGCT), we observed that the average methylation was significantly lower compared to normal tissues [2]. Our results agree with those of Guo et al. (2023) [58], who showed that HAVCR2 is hypermethylated in a considerable number of tumors, including BRCA, ESCA, HNSC, KIRP, LUAD, LUSC, LIHC, UCEC, and KIRC. We also explored the correlation between the HAVCR2 gene and the pathways, in which it takes part. We found an inducing effect on apoptosis, EMT, and ER signaling pathways. Apoptosis is programmed cell death and plays a vital role in development and homeostasis [63,64]. Its function is to eliminate any damaged cells or even cancer cells. The final result of this pathway is the induction of DNA damage and the prevention of uncontrolled proliferation [63]. Consequently, HAVCR2 acts as an inhibitor of tumor growth by activating the apoptosis pathway. Apoptosis evasion is one of the hallmarks of cancer, meaning that apoptosis occurs in each cancer cell regardless of the type [65,66]. EMT also occurs in normal occasions like embryonic development, tissue regeneration, and wound healing. Nevertheless, it can take part in tumor progression through the invasion of cancer cells, the generation of cancer cells with stem cell functions, and cancer treatment resistance. In tumor progression, EMT activates hypoxia, cytokines, and growth factors from the TME. Thus, the fact that HAVCR2 induces the EMT pathway indicates that HAVCR2 has a tumor-progressive role [67,68,69,70].

LAG3 plays a leading role in the activation of T cells. It is a biomarker present in both CD4 and CD8 T cells [71]. These can be activated and expanded by IL-12, while LAG3 expression is reduced by the blockade of IFN-γ [72]. It has been demonstrated that in the exhaustion of CD8 T cells, a variety of negative regulatory pathways act together. One of these pathways is the co-existence of inhibitory receptors, LAG3 and PD-L1. This can be verified by the analysis and the results of two papers, in which the blockade of this pathway resulted in enhanced entry of exhausted CD8+ T cells into the cell cycle. This study referred to chronic viral infections [73,74]. Nevertheless, another study confirmed that in ovarian cancer, CD8+ T cells were negatively affected by these coreceptors, and their inhibition would be of great significance for cancer therapy [73]. We also observed that LAG3 regulation is associated with DNA methylation in the promoter. These findings have been confirmed in melanoma and clear-cell renal cell carcinoma [65,66]. This LAG3 regulation has been linked with increased immune cell infiltration and enhanced OS. While most studies have found high expression of LAG3 in NSCLC or BRCA, our results showed a mild over-expression of LAG3 in LUAD and BRCA. Our findings indicated that LAG3 shows a protective role in acute myeloid leukemia (AML), ovarian cancer, and BRCA. At the same time, in bladder cancer and glioma, LAG3 demonstrated an adverse protective role. LAG3 showed comparable results to HAVCR2 regarding its correlation with apoptosis, EMT, and the ER pathway.

Furthermore, regarding PDCD1 (PD-1), its expression is typically activated in B cells, NK, CD4+, CD8+ T cells, and induced monocytes [75]. Some other ways that induce PDCD1 expression are BCR, TCR signaling, and tumor necrosis factor (TNF) [76]. PDCD1 encodes the PD-1 protein, which has a negative regulatory role in the anti-cancer T cell effector function. Once its ligand (PD-L1 [77,78]) once is expressed in the TME, it results in PD-1-mediated T cell exhaustion [79]. Anti-PD-1/PD-L1 mabs [80] are the most widely studied inhibitors in cancer immunotherapy. Our analysis indicates that PDCD1 expression was not high in the majority of the cancers, apart from KIRC. On the other hand, PDCD1 was found to have elevated expression in tumors like BRCA, KIRC, KIRP, and SKCM. At the same time, its levels were low in COAD and HNSC, among other tumor types.

In addition, we found that PDCD1 expression is positively correlated with neutrophils and SKCM. This finding is opposite to that of Miao et al. (2020) [80]. In regard to the protective role of PDCD1, we found a significant correlation between PDCD1 expression and its prognostic role in various tumors. Using PrognoScan, we found a protective role of PD1 in COAD, UVM, and SKCM, as well as an adverse protective role in BLCA, BRCA, LUAD, and OV. Comparing these results with those of Miao et al. (2020) [80], we discovered that their results in BRCA and BLCA are the opposites.

We also found that PDCD1 expression is high in the inhibition of the RTK pathway, too. Receptor tyrosine kinases (RTK) belong to a family of receptors, including epidermal growth factor receptor (EGFR), vascular epidermal growth factor receptor (VEGFR), and the insulin receptor. They are mediators of cellular proliferation and take part in functions of embryonic development and homeostasis [81]. The inhibition of this pathway means that the function of the pathway is reduced. So, by inhibiting the RTK pathway, the mediation of cellular proliferation is reduced.Dysregulation of cell proliferation is a key feature of cancer, and we can label PDCD1 as a tumor-promoting gene. Finally, we found no significant correlation between PDCD1 expression and methylation in any tumor.

CXCL13 is a member of the chemokine family, the functions of which, are key for cell survival [82]. Disruption of these molecules results in tumor growth and progression. Specifically, CXCL13, along with its receptor, CXCR5, is responsible for attracting B cells, while it also promotes the migration of specific taxis of T cells and macrophages [83]. We compared our results with other studies, but unfortunately, the majority of them were focused either on CXCL13 expression in BRCA or on the combined expression of CXC13-CXCR5 [84]. Most of the interesting findings regarding CXCL13 were in KIRC. In particular, we found that CXCL13 expression is higher in KIRC compared to the normal tissue. We also explored a positive correlation between CXCL13 and different subtypes and clinical stages in KIRC. Furthermore, we observed that lower-expression groups of CXCL13 had better DFS and PFS in this tumor. This result indicates that CXCL13 could be a candidate for therapeutic purposes. There are a lot of papers contemplating that CXCL13 can predict immune response [85]. We also investigated the prognostic role of CXCL13 in different tumors. For example, we found a protective prognostic role in BRCA and SKCM, while it showed an adverse protective role in BLCA and bladder cancer. We finally explored the correlation between CXCL13 expression and pathways. We found that CXCL13 had an inducing effect on apoptosis and an inhibitory effect on the RTK pathway. These two effects mean that CXCL13 serves as a tumor-inhibiting gene. Recent studies have revealed novel ways of CXCL13 regulation. ReIA, a subunit of the NF-κΒ family, is found to bind to the CXCL13 promoter and positively regulate CXCL13 transcription [86]. On the other hand, nuclear factor erythroid 2-related factor (NRF2) is negatively correlated with the transcription of the chemokine [78,79].

Lastly, regarding the correlation of the Tex signature with drug sensitivity, we observed significant correlations with several drugs. Notably, we found a negative correlation between Cytochalasin b and LAYN expression. We also found a strong positive correlation between LAYN expression, PHA-793887, and Belinostat. In addition, we showed that Belinostat and Avrainvilamide are negatively correlated with LAG3 expression. On the other hand, we found a mild positive correlation between Avicin D and PDCD1 expression, while HAVCR2 was negatively correlated with most drugs. We also showed that TIGIT expression is negatively correlated with PHA, BRD, Alvocidib, and Dinaciclib sensitivity. Regarding the GDSC database, our analysis highlights a high-positive correlation between Docetaxel and PDCD1, a significant negative correlation between HAVCR2 expression and TPCA-1, and a negative correlation between Docetaxel, Bleomycin and LAYN expression.

Cytochalasin B is one of the members of a family called Cytochalasins. They are mycogenic toxins that can bind to F-actin. This results in the blockade of the polymerization pathway. The effects of this blockade are visible in cellular morphology, cell division, and apoptosis [87,88,89,90,91]. The most widely studied member of this family is Cytochalasin B, which inhibits actin polymerization through binding to the F actin and interacting with proteins like CAPZA1 [92,93,94,95].

PHA-793887 is one of the newly discovered cyclin-dependent kinase inhibitors (CDKs). CDKs play a significant role in regulating the normal cell cycle [96]. Thus, they are usually deranged in most tumor cases, explaining the necessity for the development of CDK inhibitors. PHA-7889 inhibits a great variety of CDKs, like CDK1, CDK2, CDK4, CDK5, CDK7, and CDK9 [97].

Belinostat is a hydroxamic acid that generally acts as a histone deacetylation inhibitor [98]. Histone deacetylases (HDACs) function in complement to tumor growth because they induce non-normal transcription of genes that take part in significant cell features like proliferation, cell cycle regulation, and programmed cell death (apoptosis). Subsequently, there is a great need for HDAC inhibitors. These can regulate gene expression and induce chromatin relaxation, while they can also affect gene expression [99]. However, it can also act as a successful anti-tumor molecule [100]. Belinostat demonstrates nanomolar potency against class I, II, and IV HDAC isoforms. Their anti-cancer feature has been approved by the FDA for the therapy of patients with relapsed or refractory peripheral T cell lymphoma [101,102].

Docetaxel is another very potent drug that was significantly correlated with our gene signature. It is a taxoid antineoplastic molecule and it has been utilized against malignancies in the breast, prostate, and others. Docetaxel reversely binds to microtubules and inhibits cell division, while at the same time, it promotes cell death.

LAYN can play a pivotal role in the TME as it shows a negative correlation with sensitivity in Cytochalasin B and a positive correlation with sensitivity in both PHA-793887 and Belinostat. A negative correlation with Cytochalasin B indicates that it allows actin polymerization, which results in the inhibition of apoptosis and the induction of cell proliferation. On the other hand, LAYN exhibits a massive positive correlation with PHA-793887 and Belinostat. Both drugs have an anti-cancer feature, as they inhibit cell proliferation or cell division and promote cancer cells to the apoptosis pathway. LAG3 exhibited a significant negative correlation with Belinostat, suggesting that it may be a future potent target for developing a new generation of drugs.

Similar results were found for HAVCR2 and TIGIT, too. TIGIT is one of the most potent anti-cancer targets using ICIs. In addition, PDCD1 demonstrated a very high positive correlation with sensitivity in docetaxel, suggesting that this drug could be used successfully to treat cancer, alone or in combination with ICIs like pembrolizumab and nivolumab. Since docetaxel binds to the microtubules, it is a promising intracellular agent.

On the other hand, LAYN showed a strong negative correlation with sensitivity in Docetaxel, indicating that LAYN may have better results as an extracellular marker than an intracellular one. However, these are hypotheses we make according only to the drug sensitivity of these genes. We are aware that a plethora of results both in vivo and ex vivo indicate the efficacy of the genes in drugs, like epigenetic modulations, mutations, the stage of the tumor in each patient, and the genetic background of each patient.

One limitation of our study is the absence of in vitro or in vivo experiments to validate our findings and enhance their significance.

## 5. Conclusions

In conclusion, we explored in depth the correlation of gene expression patterns in Tex, gene mutation, gene methylation, patient survival, immune infiltration, and pathway activity in pan-cancer. We also underlined the potent effect of this signature on anti-cancer drug sensitivity. Overall, our findings highlight the importance of reviving exhausted T cells to enhance the treatment efficacy to significantly boost anti-tumor immunity and achieve better clinical outcomes.

## Data Availability

Genomic data were extracted from TCGA (https://portal.gdc.cancer.gov/, accessed on 1 March 2024)). Small molecule drugs’ data were extracted from GDSC (https://www.cancerrxgene.org/, accessed on 1 May 2024)) and CTRP (https://portals.broadinstitute.org/ctrp/, accessed on 1 May 2024)). Immunogenomic data analysis was performed using ImmuCellAI (http://bioinfo.life.hust.edu.cn/ImmuCellAI#!/, accessed on 1 May 2024)).
